# The relations between business model efficiency and novelty, and outcome while accounting for managed competition contract: a quantitative study among Dutch physiotherapy primary healthcare organisations

**DOI:** 10.1186/s12913-022-08383-7

**Published:** 2022-08-03

**Authors:** Rutger IJntema, Di-Janne Barten, Hans Duits, Brian Tjemkes, Cindy Veenhof

**Affiliations:** 1 Research Group Financial-Economic Advise on Innovation, HU University of Applied Sciences Utrecht, Heidelberglaan 15, Utrecht, 3584 CS the Netherlands; 2 Department of Rehabilitation, Physical Therapy Science and Sports, Brain, Centre Rudolf Magnus, Utrecht University, University Medical Centre Utrecht, Utrecht, the Netherlands; 3 Institute Finance and Accounting, HU University of Applied Sciences Utrecht, Utrecht, the Netherlands; 4 Department of Management and Organisation Studies, VU University Amsterdam, Amsterdam, the Netherlands

**Keywords:** Physiotherapy, Physical therapy, Business model efficiency, Business model novelty, Managed competition contract, Primary healthcare, Performance, Outcomes

## Abstract

**Background:**

Since 2006, business principles have been introduced to foster efficient healthcare by way of managed competition. Managed competition is expressed by a contract between a health insurer and a physiotherapy primary healthcare organisation (PTPHO). In such a managed environment, PTPHOs have to attain treatment service quality and financial PTPHO-centred outcomes Research shows that business model designs may enhance organisation-centred outcomes. A business model is a design (efficiency or novelty) of how a firm transacts with customers, partners, and vendors; how it connects with markets. However, research on managed competition contract and business model designs, in relation to PTPHO-centred outcomes is new to the healthcare literature. PTPHOs may not know how business model designs enhance outcomes. This study aims to delineate the relations between business model efficiency and novelty, and PTPHO-centred outcomes, while accounting for managed competition contract in Dutch healthcare.

**Methods:**

A quantitative cross-sectional design was adopted. Using a questionnaire, the relations between managed competition, business model efficiency and novelty, and PTPHO-centred outcomes were investigated among PTPHO managers (*n* = 138). Theory-based expectations were set up and multiple linear regression analyses were applied.

**Results:**

Managed competition and business model efficiency show no relation with PTPHO-centred outcomes. Moderation of the business model efficiency and PTPHO-centred outcomes relation by managed competition contract is not detected. Business model novelty shows a positive relation with PTPHO-centred outcomes. Moderation of the business model novelty and PTPHO-centred outcomes relation by managed competition contract is found.

**Conclusions:**

There seem to be positive relations between business model novelty and PTPHO-centred outcomes on its own and moderated by managed competition contract. No relations seem to exist with business model efficiency. This implies that the combination of persistent use of health insurer-driven managed competition contracts and a naturally efficient PTPHOs may have left too few means for these organisations to contribute to healthcare reforms and attain PTPHO-centred outcomes. Organisation-driven innovation could stretch system-level regulations and provide room for new business models. Optimising contracts towards organisation-driven healthcare reform, including novelty requirements and corresponding reimbursements is suggested. PTPHO managers may want to shift their attitudes towards novel business models.

**Supplementary Information:**

The online version contains supplementary material available at 10.1186/s12913-022-08383-7.

## Background

### Managed competition

Managed competition was introduced in healthcare systems by governments and has emerged since the 1990s, at varying speeds and levels of success, in countries such as the United Kingdom, Germany, the United States and the Netherlands [1, 2, 3]. Managed competition uses rules to instil efficiency in healthcare systems, that is, higher quality, lower costs, value for money, and achievement of standardised products and services [4, 5]. With managed competition, business principles have been introduced in the healthcare markets [6, 7]. For example, within healthcare markets, managed competition stimulates health insurers to compete for patients and contract healthcare organisations like primary healthcare organisations. Concurrently, healthcare organisations vie for contracts with health insurers. Likewise, patients are encouraged to select a health insurer and healthcare organisations of their choice [8, 9, 10]. However, many preconditions for managed competition are not fulfilled. An example is that the competition activities of health insurers, healthcare organisations and patients are rather weak, and health care costs are still rising so far [8, 11]. Another example is that negotiation power is not equally divided between managed competition participants [6–, 12, 13, 14].

### Healthcare organisation context

The effects of managed competition depend to a significant extent on the context and measurement of the organisation providing the healthcare [5–, 15, 16, 17, 18]. However, managed competition effects may not yet be known because of a paucity of healthcare organisation context-specific empirical evidence. If such evidence is available, most discussions revolve around the hospital context instead of the primary healthcare organisation context [18, 19]. The managed competition debate is about the degree of healthcare system-level rules versus the degree of healthcare organisation-level competition [7, 18]. Rules-based efficient, standardised products and services may hinder healthcare organisation context-specific responses and lead to organisational stagnation instead of innovation. Although proponents of managed competition mention that the competition urges healthcare organisations to boost their efficiency, the opponents state that the competition between healthcare organisations is ineffective [20]. Among managed competition participants the healthcare providers are most dissatisfied. The healthcare providing organisations need better compensation for evidence-based innovations [6, 21]. Turning attention to context-specific concerns may be necessary to further managed competition [5, 6, 7, 22, 23].

### Managed competition contract and organisation-centred outcomes

A contract between a healthcare organisation and a health insurer that specifies intended outcomes in return for financial reimbursement is often how managed competition is expressed [10, 15, 16]. The healthcare management literature seems inconclusive on the relation between managed competition contract and organisations-centred outcomes. On one hand the healthcare management literature suggests that healthcare organisations that do the best job of improving treatment service quality, cutting cost, and satisfying patients, are rewarded with more patients and revenue [4, 5, 15, 18, 20, 21, 24]. Also, in an empirical paper on a contract pilot in dental practices an increase in registered patients, a reduction in volumes of treatments, increased financial income and changes in patient satisfaction was seen [25] In a conceptual paper, Boone and Schotmüller [14] suggest that managed competition contracts reduce healthcare organisation costs. In a health policy paper Shmueli et al. [13] mention that contracting may lead to increased patient satisfaction. On the other hand, healthcare management literature suggests that no healthcare system thus far has achieved the necessary mix of incentives for healthcare organisations. Furthermore, to achieve optimal contracts the tension between rules-based standardisation and (business model) innovation must be reconciled [16]. Little empirical basis for treatment service quality outcomes in relation to managed competition contract is found within healthcare organisations [15, 26].

### Dutch PTPHO context

To advance understanding om managed competition contracts, an exemplary example of managed competition in a specific context is found in the Dutch healthcare market. First, in 2006, the Health Insurance Act and the Health Care Market-Regulation Act were introduced as a legislative framework, which formed the foundation for managed competition in the Netherlands [9, 27]. Internationally, the Dutch healthcare market may have the longest experience with managed competition [8]. Second, Dutch physiotherapy primary healthcare organisations (PTPHO) pioneered managed competition during a government-led experiment from 2005 to 2007 [28]. Particular characteristics of Dutch PTPHOs are that these organisations provide “services for individuals and populations to develop, maintain and restore maximum movement and functional ability throughout the lifespan” within their local context [29]. Of these approximately 10,000 PTPHOs 96% have less than 10 persons working. This means that PTPHOs are small businesses. Furthermore, PTPHOs are private businesses with limited resources to respond to managed competition [30, 31, 32]. Qualified and multitasking PTPHO managers with context-specific knowledge that delegate to the employed physiotherapist, administrative staff, and hired external professionals lead these organisations [33]. Although theory on managed competition contract in relation to organisation-centred outcomes seems inconclusive, in the Dutch context a health insurer has power by offering contracts to PTPHOs that include high efficiency requirements and corresponding reimbursements. Recorded requirements are, for example, a Dutch PTPHOs receives the highest reimbursement level if they participate in a national treatment efficiency index for specified patient populations. As well, if they report on improvements in efficiency indicators at healthcare system level and external audits [34, 35]. This means that a PTPHO that complies with the highest contract requirements receives the highest reimbursement level, resulting in increased revenue. In summary, although theory seems inconclusive, based on the daily reality of contract requirements and corresponding reimbursement for PTPHOs, *it could be expected that managed competition contract is positively related to PTPHO-centred outcomes.*

### Dutch context and PTPHO-centred outcomes

Organisation-centred outcomes in the specific context of the Dutch PTPHOs are described in a review by IJntema et al. [15]. The review indicates treatment service quality and financial results as the main PTPHO-centred outcomes. Treatment service quality is related to the overall accuracy of the care-providing organisation, like medical diagnoses, standards, guidelines, protocols, and a variety of treatment options. Also, how the care delivered by the organisation is perceived by the patients. Financial outcomes are expressed in for example revenue, cost, sales growth, revenue growth, and profit. Other healthcare policy and management studies indicate similar outcomes [10, 27–, 36, 37, 38].

### Business model designs

Because business principles are involved in managed competition contracts, this study draws from the business model lens delineated in business model theory. A business model is a design of how a focal firm transacts with customers, partners, and vendors; that is, how it chooses to connect with (healthcare) markets [39]. For example, a business model may link administration, finances, monitoring and supervising staff, partners, and overseeing healthcare market developments [15]. Another example is how a focal PTHPO connects with general practitioners, other healthcare professionals, and a health insurer to start a multi-professional primary healthcare programme, including a cost and earnings model.

The business model literature refers to business model efficiency and novelty designs [40]. Business model efficiency refers to the measures an organisation takes to achieve efficient transactions with its customers, partners and vendors [39]. Within the context of PTPHOs, the efficiency design pertains to accurately sharing patient-related and financial data information with patients, healthcare providers, and health insurers to ensure informed decisions. It also refers to the use of information, treatment services, and material that is verifiable and evaluable. In the context of PTPHOs business model efficiency means employing efficient transactions to reduce transaction costs [33, 40]. In line with business model theory, *it could be expected that business model efficiency is positively related to PTPHO-centred outcomes.*

Business model novelty refers to the conceptualisation and adoption of new ways of transactions between a focal PTPHO and its customers, partners and vendors [39]. Business model novelty has shown to be a pre-condition for organisation-centred outcomes [41]. In the PTPHO context, this means the organisation exploits different transactions compared to other competitors [41, 42]. Examples are new treatment services and brand-new ways of information administration and exchange, like real-time patient-related and financial data information, and connecting to untried stakeholders to create new opportunities. The need for business model novelty is further expressed in the healthcare management literature. For example, new business models have stimulated new treatment services like e-Exercise by physiotherapists [43]. Also, with a novelty design barriers to innovation adoption by organisations could be resolved [44]. In addition, novel models may enhance a PTPHO to modify treatment content, change the order or timing of services and organise multi-professional collaboration [45]. In line with current business model theory, *it could be expected that business model novelty is positively related to PTPHO-centred outcomes.*

### Moderating influence of managed competition contract

Business model designs may be needed to attain managed competition contract requirements and PTPHO-centred outcomes [6, 15, 39]. Several studies recommend that context-specific innovation-driving requirements, and treatment service quality and financial organisation-centred outcomes may need to be included in contracts [10, 16, 23, 27]. However, the healthcare management literature lacks research on the moderating influence that a managed competition contract can have on the relation of business model efficiency and novelty, with PTPHO-centred outcomes [6, 20, 24].

Managed competition contract and business model efficiency might both enable a PTPHO to foster efficiency and to attain PTPHO-centred outcomes. One might expect that compliance with the highest contract requirements enhances the relation between business model efficiency and PTPHO-centred outcomes. In contrast, non-compliance with the highest contract requirements may hinder that relation, resulting in fewer revenues. *It could be expected that the relation between business model efficiency and PTPHO-centred outcomes is moderated by managed competition contract such that when the contract requirements are the highest, the relation is positive, and when the contract requirements are not the highest, the relation is negative.*

Because of a difference in organisation- level requirements and healthcare system-level requirements, contract requirements may not match the needs at the organisational level [46]. The healthcare management literature suggests that although business model novelty may enable organisation-centred outcomes, a managed competition contract may hinder PTPHO-centred outcomes because the highest contract requirements focus on rewarding efficiency, not novelty [23]. For example, although potentially helpful, a novel idea like preventative care instead of curative care may not be reimbursed based on managed competition contract efficiency requirements. Underinvestment in novelty by the health insurer may result in a limitation of innovation and decreased PTPHO-centred outcomes [10, 21]. Investments in novelty outside the highest contract requirements may well lead to PTPHO-centred outcomes because organisations may be more flexible to assert themselves through innovative ideas [10]. *It could be expected that the relation between business model novelty and PTPHO-centred outcomes is moderated by management competition contract, such that when the contract requirements are the highest the relation is negative and when the contract requirements are not the highest the relation is positive.*

The business model and healthcare management literature lack comprehensive insights into business model designs related to PTPHO-centred outcomes, while accounting for managed competition contract [6, 36, 37, 47, 48]. Furthermore, the existing body of knowledge on business model efficiency and novelty has evolved outside the primary healthcare context. As a consequence, PTPHO managers may not know which business model design (efficiency or novelty) explains their organisation’s outcomes. This study aims to delineate the relations between business model efficiency and novelty, and PTPHO-centred outcomes, while accounting for managed competition contract in Dutch healthcare.

## Methods

### Data collection and sample

A quantitative cross-sectional design was adopted and applied to the Dutch PTPHO context. Data were collected during 2 months from August 2020 by an online self-administered questionnaire which was sent to Dutch managers responsible for PTPHO-centred outcomes. Via an open online announcement in regular newsletters of Dutch physiotherapy and healthcare associations, and public and private physiotherapy networks, the PTPHO managers were invited to voluntarily and anonymously respond to the questionnaire.

### Illustration of expected relations

**Fig. 1:**
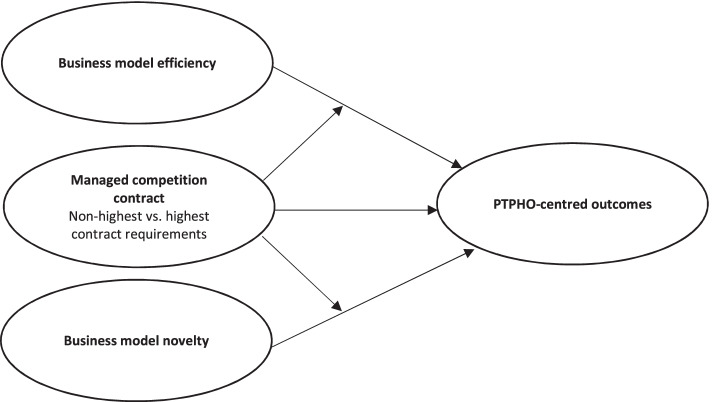
Illustration of expected relations

Figure 1 illustrates the relations that could be expected between business model efficiency and novelty, and PTPHO-centred outcomes while accounting for managed competition contract.

### Measurement

As a result of a search in healthcare and management literature instruments were selected to compose a questionnaire. The starting point was to select only existing and validated instruments for measuring the introduced constructs. These instruments have already proven their worth in contexts other than the PTPHO context.

### PTPHO-centred outcomes

To measure PTPHO-centred outcomes, the perceived organisational performance scale designed for small and medium-sized businesses used by Hung and Chiang [49], was selected. This instrument was previously used in small and medium enterprises. The six items were evaluated on a five-point Likert scale ranging from ‘much lower’ (1) to ‘much higher’ (5). The scale was calculated by the mean value of all items.

### Managed competition contract

Managed competition contract measurement was based on publicly available contract terms of Dutch health insurers. Because the terms showed a split in contract requirements including corresponding reimbursements, managed competition contract was dichotomised by ‘non-highest contract requirements’ (0) or ‘highest contract requirements’ (1).

### Business model designs

The scale for business model efficiency was originally tested by Zott and Amit [39] in a population of publicly listed entrepreneurial firms. That scale included 13 items that were evaluated on a four-point Likert scale: ‘strongly disagree’ (0), ‘disagree’ (0.25), ‘agree’ (0.75), ‘strongly agree’ (1). The scale was calculated by the mean value of all items. The scale for business model novelty was a nine-items scale used by Guo et al. [42], that was tested in small- and medium enterprises was adopted. The items were evaluated on a five-point Likert scale ranging from ‘strongly disagree’ (1) to ‘strongly agree’ (5). The scale was calculated by the mean value of all items.

### Control variables

Control variables were included that are common in management research, or relevant in the PTPHO context, which potentially confound the relations illustrated in Fig. 1 [50]. PTPHO manager control variables were gender, age and education degree (that is, bachelor or master degree). Organisational control variables were organisation type (that is, private ownership or shareholders involved), number of departments (one or more than one departments), number of employees (full-time equivalent) and specialised physiotherapist employed by the PTPHO (no or yes). The latter is relevant for the Dutch context. In the Netherlands, a specialised therapist (paediatrics, sports, mental health, etc.) applies skills that may influence business model efficiency or novelty of a PTPHO. In addition, a specialised therapist may receive a higher financial reimbursement than a non-specialised physiotherapist, which may influence PTPHO-centred outcomes. In Table 1 an overview of the measurement instruments is shown.

**Table 1:** Overview of the measurement instruments

Variable	Short description of the study’s variables	Instrument	Authors, year	Context	Scale
PTPHO-centred outcomes	Outcomes related to the overall accuracy of the care, like medical diagnoses, standards, guidelines, protocols, and variety of treatment options Outcomes expressed in cost, sales growth, revenue growth and profit	Small/medium enterprise performance	Hung and Chiang, 2010 [49]	Small/Medium enterprises	5 point Likert
Managed competition contract	Contract between health insurer and healthcare organization that specifies intended outcomes in return for financial reimbursement	–	–	PTPHO context	Dichotomised
Business model efficiency	Measures an organisation takes to achieve efficient transactions with its customers, partners and vendors	Business model efficiency	Zott and Amit, 2007 [40]	Entrepreneurial firms	5 point Likert
Business model novelty	Conceptualisation and adoption of new ways of transactions between an organisation and its customers, partners and vendors	Business model novelty	Guo et al., 2017 [42]	Small/Medium enterprises	5 point Likert
Control variables	Variables that potentially confound the relations under study	–	Atinc and Simmering, 2021 [50]	Large, Medium and small enterprises	Dichotomised Numerical

All measurement instruments adopted were originally developed in English, so for the current study, the instruments were translated into Dutch. The selection of measurements was followed by pre-testing the online questionnaire by two PTPHO managers to gain insight into potential technical failures, response time, and the face validity for the PTPHO context. The comments of the managers were collected and discussed between the first and second authors of the current article. Were needed improvements to the questionnaire were made.

### Preparation for statistical analyses

Preparations for statistical analyses were made because the applied measurement instruments were not tested in advance on psychometric properties like the reliability or appropriateness for use in the particular PTPHO setting. The reliability of the scales was calculated by determining Cronbach’s alpha and factor loadings. The minimum level of Cronbach’s alpha was > 0.7 for all instruments which indicates appropriate internal consistency [51]. Exploratory factor analysis was conducted for each scale item with an applied factor loadings cut-off point of > 0.6 which indicates appropriate internal reliability [51]. The exploratory factor analysis revealed that PTPHO-centred outcomes contained two dimensions: treatment service quality and financial. Because managed competition contract concerned one question, no exploratory factor analysis nor Cronbach’s alpha was applied. More information about the scale, scale-items, factor loadings and Cronbach’s alpha is shown in Additional file 1. Last, a Pearson correlation test was used to assess correlations between all variables and to check for collinearity. No indication of a strong or very strong correlation was detected based on a < 0.6 cut-off point [52]. The result of the correlation test is shown in Additional file 2. To describe the significance level for each correlation, a cut-off point *p* value < 0.05 was applied.

### Statistical analysis

**Fig. 2:**
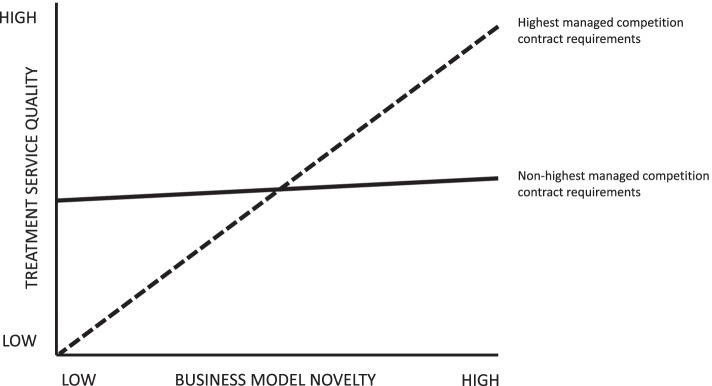
Illustration of business model novelty – treatment service quality relation moderated by managed competition contract

**Fig. 3:**
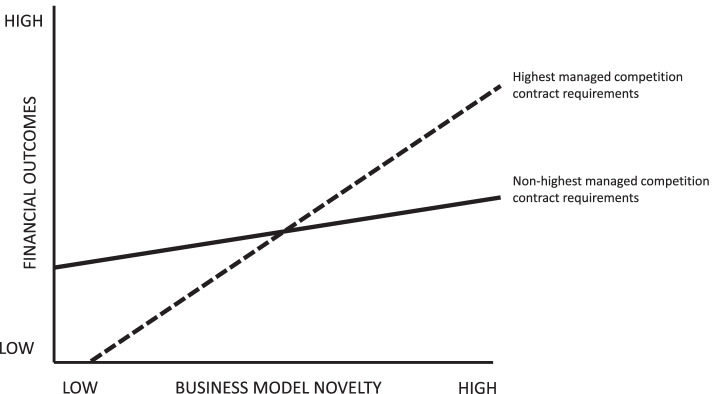
Illustration of business model novelty – financial outcomes relation moderated by managed competition contract

Descriptive analyses were performed on all outcomes by describing the number (percentage), mean of sample, and standard deviation when applicable. The statistical significance of the sample concerning the response rate of PTPHO managers including a confidence interval was calculated as well. Multiple linear regression analyses were used to delineate the relations between business model efficiency and novelty, and both treatment service quality and financial PTPHO-centred outcomes, while accounting for managed competition contracts. Because the concepts were new to the PTPHO context, all scale items were included based on theory, rather than techniques like stepwise analysis. All variables included in the study were checked in advance on criteria for multiple linear regression conditions by analysis of the mean, median, standard deviation, maximum, minimum, skewness and kurtosis. Furthermore, a variance inflation factor (VIF) test was applied with a < 2.5 cut-off point that was set for multicollinearity. Various theory-based models, were fitted and compared with each other (Tables 3 and 4). Because moderation is analysed, to alleviate possible multicollinearity, age, number of departments, number of employees, business model efficiency and novelty, were mean-centred [53]. To describe the significance level for each variable within a model, a cut-off point *p* value < 0.05 was applied. Also, the adjusted *R*^2^ was calculated to describe the explanatory power of each constructed model. Statistical analysis was performed by R version 4.1.0. Finally, possible moderation by managed competition contract was illustrated (Figs. 2 and 3).

## Results

### Descriptive characteristics

Table 2 shows the descriptive characteristics of the PTPHO managers, the PTPHOs, and the dependent and independent variables. The sample included 138 valid questionnaires returned by the participants. The sample comprises small businesses with a mean number of 6.65 (sd 5.6) employees full-time equivalent. Furthermore, the majority (77%) of the PTPHOs represent a private ownership type of organisation and the vast majority (89%) of the PTPHOs employ one or more specialised therapists. Managed competition contract highest contract requirements comprise 45% of the sample.

**Table 2:** Descriptive characteristics

	N (percent)	Mean of sample	Standard deviation
**PTPHO manager characteristics**			
Gender			
*Male*	76 (55)		
*Female*	62 (45)		
Age		50.50	10.44
Education			
*Bachelor*	85 (62)		
*Master*	53 (38)		
**PTPHO characteristics**			
Organisation type			
*Private ownership*	106 (77)		
*Shareholders*	32 (33)		
Number of departments			
*One*	59 (43)		
*More than one*	79 (57)		
Number of employees (full time equivalent)		6.65	5.58
Specialised therapist			
*No*	15 (11)		
*Yes*	123 (89)		
**Dependent variables**			
PTPHO-centred outcomes – treatment service quality (range 1–5)		3.18	0.40
PTPHO-centred outcomes – financial (range 1–5)		3.34	0.69
**Independent variables**			
Business model efficiency (range 0–1)		0.74	0.12
Business model novelty (range 1–5)		3.56	0.63
Managed competition contract			
*Non-highest contract requirements*	76 (55)		
*Highest contract requirements*	62 (45)		
**Sample size: 138**			

All variables included in the study met conditions for normal distribution. In addition, with 138 valid questionnaires returned, based on a 10,000 PTPHO population, the margin of error is 8.3% (not shown in Table 2).

### Delineation of relations

Table 3 shows the relations between business model efficiency and novelty and PTPHO-centred outcomes treatment service quality while accounting for managed competition contract controlled for potential confounders.

**Table 3:** Regression analysis on PTPHO-centred outcomes - treatment service quality

	***Model***				
	***1***	***2***	***3***	***4***	***5***
Control variables	ß	ß	ß	ß	ß
Gender	0.05	0.05	0.05	0.05	0.06
Age	0.00	0.00	0.00	0.00	0.00
Education degree	−0.01	− 0.01	− 0.02	− 0.02	− 0.03
Organisation type	0.01	0.01	− 0.04	0.01	− 0.09
Number of departments	− 0.03	− 0.04	−0.06	− 0.04	−0.02
Number of employees (full time equivalent)	0.00	0.01	0.00	0.00	−0.01
Specialised therapist	0.11	0.11	0.10	0.12	0.12
Expected relations	ß	ß	ß	ß	ß
Managed competition contract	0.04			0.03	0.09
Business model efficiency		0.18		0.06	
Business model novelty			0.18***		0.03
Business model efficiency*Managed competition contract				0.26	
Business model novelty*Managed competition contract					0.38***
*R*^2^_adj_	−0.03	− 0.03	0.04	−0.04	0.11

*n* = 138

*ß* estimate

***P < 0.001

The relations between business model efficiency and novelty and financial PTPHO-centred outcomes while accounting for managed competition contract controlled for potential confounders are delineated in Table 4.

**Table 4:** Regression analysis PTPHO-centred outcomes - financial

Estimates / Model	Model				
	***1***	***2***	***3***	***4***	***5***
Control variables	ß	ß	ß	ß	ß
Gender	0.13	0.12	0.11	0.13	0.14
Age	−0.01	−0.01	0.00	− 0.01	0.00
Education degree	0.12	0.11	0.09	0.11	0.09
Organisation type	−0.20	−0.21	− 0.27	−0.20	− 0.31**
Number of departments	−0.13	− 0.13	−0.17	− 0.14	−0.13
Number of employees (full time equivalent)	0.04***	0.04***	0.03**	0.04***	0.03**
Specialised therapist	0.52**	0.50**	0.47**	0.53**	0.52***
Expected relations	ß	ß	ß	ß	ß
Managed competition contract	−0.16			−0.16	−0.11
Business model efficiency		0.01		−0.06	
Business model novelty			0.25**		0.09
Business model efficiency*Managed competition contract				0.22	
Business model novelty*Managed competition contract					0.40**
*R*^2^_adj_	0.11	0.11	0.15	0.10	0.18

*n* = 138

*ß* estimate

*P < 0.05; **P < 0.01; ***P < 0.001

### Managed competition contract related to PTPHO-centred outcomes

Managed competition contract shows no significant relation with treatment service quality nor financial PTPHO-centred outcomes (Models 1, Tables 3 and 4).

### Business model efficiency related to PTPHO-centred outcomes

Based on models 2 (Tables 3 and 4), business model efficiency shows no significant relation with treatment service quality and financial PTPHO-centred outcomes.

### Business model novelty related to PTPHO-centred outcomes

Model 3 in Table 3 (*R*^2^_adj_ 0.04) shows a significant positive relation between business model novelty and treatment service quality PTPHO-centred outcomes (ß 0.18, *p* < 0.001). Also, Model 3 in Table 4 shows a significant positive relation between business model novelty and financial PTPHO-centred outcomes (ß 0.25, *p* < 0.01, *R*^2^_adj_ 0.15).

### Relation between business model efficiency and PTPHO-centred outcomes moderated by managed competition contract

Models 4 (Tables 3 and 4) also show results with managed competition contract as a possible moderator of the relation between business model efficiency, and treatment service quality and financial PTPHO-centred outcomes. No significant moderation of the business model efficiency and treatment service quality PTPHO-centred outcomes relation is detected. Likewise, no significant moderation of the business model efficiency and financial PTPHO-centred outcomes relation is found.

### Relation between business model novelty and PTPHO-centred outcomes moderated by management competition contract

Model 5 in Table 3 shows a significant moderation of the business model novelty and treatment service quality PTPHO-centred outcomes relation (ß 0.38, *p* < 0.001, *R*^2^_adj_ 0.11). Furthermore, Model 5 in Table 4 shows a significant moderation of the business model novelty and financial PTPHO-centred outcomes relation (ß 0.40, *p* < 0.01, *R*^2^_adj_ 0.18).

Figure 2 illustrates how managed competition contract may moderate the business model novelty and treatment service quality PTPHO-centred outcomes relation. The highest managed competition contract requirements seems to entail a stronger positive relation with the business model novelty and treatment service quality PTPHO-centred outcomes relation than the non-highest contract requirements.

Figure 3 illustrates a detailed insight on how managed competition contract may moderate the business model novelty and financial PTPHO-centred outcomes relation. The figure shows that the highest managed competition contract requirements may entail a stronger positive relation with the business model novelty and financial PTPHO-centred outcomes relation than the non-highest contract requirements.

## Discussion

This study shows that, rather than efficiency, both business model novelty on its own and business model novelty moderated by managed competition contract show a significant positive relation with PTPHO-centred outcomes treatment service quality and financial. The reason that business model efficiency may not have significant relations could be that persistent health insurer-driven use of managed competition contracts have pushed PTPHOs to the limits of their business model efficiency possibilities [7]. Besides, PTPHOs are considered micro-businesses that are naturally efficient, regardless of contract requirements [54]. The combination of managed competition contracts and naturally efficient PTPHOs may explain why it is not relevant for these organisations to strive for business model efficiency in relation to PTPHO-centred outcomes [5].

The positive relation between business model novelty and PTPHO-centred outcomes, while accounting for managed competition contract may as well be explained by the combination of persistent health insurer-driven use of managed competition contracts and naturally efficient PTPHOs. The significant result is notable because it goes against the expectation that the highest managed competition contract requirements would hinder PTPHO-centred outcomes. In addition, the healthcare management literature does not seem to corroborate the positive relation. For example, Mühlbacher et al. [10] suggest that, outside the highest contract requirements, PTPHOs may be more flexible by asserting themselves through innovative ideas. Nevertheless, one could reason that PTPHOs that do not comply with the highest contract requirements may have less (financial) scope to invest in business model novelty because they receive less reimbursement. The highest contract reimbursement level may create (financial) room for a PTPHO to invest in novelty rather than business model efficiency.

From a theoretical perspective this study may have detected a trade-off between managed competition contract compliance at the PTPHO sector level, and the focal PTPHO business model design (that is, efficiency or novelty). In other words, this study sheds light on insurer-driven healthcare reform contrasted by organisation-driven healthcare reform [7]. On one side, insurer-driven reforms have focussed on fostering efficient healthcare by rewarding healthcare providers that do the most efficient job. However, the compound of persistent health insurer-driven use of managed competition contracts and a naturally efficient PTPHO sector may have left too few means for the physiotherapy sector to contribute to healthcare reforms. On the other side, at the level of the focal PTPHO, instead of efficiency, both business model novelty and business model novelty moderated by managed competition contract may enhance treatment service quality and financial PTPHO-centred outcomes. To achieve the best possible outcomes, it seems that an above-average business model novelty effort by the PTPHO is needed in combination with the highest contract requirements (Figs. 2 and 3). A trade-off between regulator-driven versus organisation-driven reform seems also evident in research outside the PTPHO context. For example, Uber introduced innovative services in several regulated markets enhanced by innovative internet platform technology business models. These services were so innovative that regulators, after initial resistance, had to adapt to Uber’s taxi drivers [55]. An example from the highly regulated financial sector is that financial entrepreneurs are cautiously allowed by regulators to test organisation-driven innovations with fewer regulatory constraints and less risk of enforcement action [56]. Last, solar industry research suggests that although not guaranteed, organisation-driven innovation could stretch existing system-level regulations and provide room for new business models [57].

The exchange between insurer-driven healthcare reform contrasted by organisation-driven healthcare reform may have three practical policy implications that may need in-depth consideration. First, efficiency contract requirements may need to be alleviated and novelty requirements introduced. For example, PTPHOs could receive reimbursement for new ideas, like bonding with patients, partners and vendors in novel ways. Second, because health insurers and PTPHO managers may each have their unique perspectives, literature suggests to optimise managed competition contracts associated with insurer-driven perspectives towards provider-driven viewpoints. For example, optimisation of contracts could be realised by balancing efficiency and innovation requirements [7, 16, 27]. Third, apart from the current state of managed competition contracting, PTPHO-managers may want to shift their attitudes toward new routines [58, 59], implementing E-health services [60], and introducing novel business models, including treatment service quality and financial outcomes [61].

### Strengths and limitations

Findings should be generalised with caution. Because the study context of small business PTPHOs has a novel character, pioneering research may run the risk of having used a unique sample. To the knowledge of the authors, this is the first study to focus on business model efficiency and novelty concerning physiotherapy organisation-centred outcomes while accounting for managed competition contract in the primary healthcare context.

The applied measurement instruments, originally developed and validated in small business contexts, are not tested in advance on psychometric properties like the reliability or appropriateness for use in the particular PTPHO setting. However, the sample of PTPHOs comprises small businesses with a mean number of 6.65 (sd 5.6) employees full-time equivalent. This is similar to the population of Dutch PTPHOs [32]. In addition, the instruments, are pre-tested by PTPHO managers. Calculations indicated appropriate scale and internal reliability. As a result, measurement instruments manageable for PTPHO managers are adopted, developed and applied.

Another important aspect of this study is that the explanatory power of the calculated models could be regarded low, as shows by the *R*^2^_adj_ of model 5 in Table 3 (0.11) and model 5 in Table 4 (0.18). This may be because a PTPHO is a multidimensional context and it may be hard to cover relevant elements in one questionnaire [5, 20, 58, 62]. For example, other potential variables, like organisational learning [63] and market orientation [64] that justify the complexity of the PTPHO context, may have been neglected.

Despite limitations, knowledge is added to the business model and healthcare management body of knowledge by researching both business model efficiency and novelty within a PTPHO context, and the moderating role of managed competition contract.

Because this study is cross-sectional by design, it is not possible to claim causal relations between the business model design, managed competition contract and outcome variables. Based on the current study, a longitudinal design could be used in future research to investigate causal relations between these variables. Furthermore, observational studies could shed light on context specific measures. Because Likert scales with perceived questions were used, future studies could apply objective measures, like financial data.

## Conclusions

There seem to be positive relations between business model novelty and PTPHO-centred outcomes on its own and moderated by managed competition contract. No relations seem to exist with business model efficiency. This implies that the combination of persistent use of health insurer-driven managed competition contracts and a naturally efficient PTPHOs may have left too few means for these organisations to contribute to healthcare reforms and attain PTPHO-centred outcomes. Organisation-driven innovation could stretch system-level regulations and provide room for new business models. Optimising contracts towards organisation-driven healthcare reform, including novelty requirements and corresponding reimbursements is suggested. PTPHO managers may want to shift their attitudes towards novel business models.

## Supplementary Information

**Additional file 1.** Scale, items, factor loadings and Cronbach’s alpha.

**Additional file 2.** Pearson correlation for all regression variables.

## Data Availability

The data that support the findings of this study are available from PTPHO managers but restrictions apply to the availability of these data, which were used under license for the current study, and so are not publicly available. Data are however available from the authors upon reasonable request and with permission of PTPHO managers.

## Abbreviation

PTPHO: physiotherapy primary healthcare organisation
