# Supplementary material for: The relations between business model efficiency and novelty, and outcome while accounting for managed competition contract: a quantitative study among Dutch physiotherapy primary healthcare organisations
Source: BMC Health Serv Res. 2022 Aug 3;22:990. doi: 10.1186/s12913-022-08383-7 (PMC9351119; doi:10.1186/s12913-022-08383-7)
Supplement: Supplementary file 1 — Additional file 1. Scale, items, factor loadings and Cronbach’s alpha. [file 12913_2022_8383_MOESM1_ESM.docx]

| **Scale** | **Items** | **Factor loadings** | **Cronbach’s alpha** |
| --- | --- | --- | --- |
| **PTPHO-centred outcomes**  **financial** | *To what extent does your organisation attain its expected results in terms of:* |  | 0.91 |
|  | Treatment service sales growth | 0.88 |  |
|  | Revenue growth | 0.96 |  |
|  | Net profit margin | 0.89 |  |
| **PTPHO-centred outcomes**  **treatment service quality** | *To what extent does your organisation attain its expected results in terms of:* |  | 0.78 |
|  | Quality of treatment service | 0.85 |  |
|  | The variety of treatment services offered | 0.79 |  |
|  | Patient satisfaction | 0.80 |  |
| **BM efficiency** | The business model enables stakeholders and patients to make informed decisions | 0.68 | 0.76 |
|  | Transactions are transparent: flows and use of information, treatment services, and materials can be verified | 0.73 |  |
|  | As part of transactions, information is provided to stakeholders and patients | 0.63 |  |
|  | Access to a large range of treatment services, information, stakeholders, and patients is provided | 0.64 |  |
|  | The business model enables fast transactions | 0.66 |  |
|  | The business model, overall, offers high transaction efficiency | 0.62 |  |
| **BM novelty** | *Our business model:* |  | 0.87 |
|  | Offers new combinations of treatment services and information | 0.76 |  |
|  | Attracts a lot of new healthcare suppliers and partners | 0.66 |  |
|  | Bonds stakeholders and patients together in novel ways | 0.76 |  |
|  | Links stakeholders and patients to transactions in novel ways | 0.74 |  |
|  | *We frequently introduce new:* |  |  |
|  | Ideas and innovation into our business model | 0.77 |  |
|  | Operational processes, routines, and norms into our business model | 0.66 |  |
|  | We are pioneers of the business model | 0.75 |  |
|  | Overall, our business model is novel | 0.64 |  |
| **Managed competition contract** | *Does your PTPHO have one or more contracts with a health insurer:* |  |  |
|  | The contract meets the highest contract requirements of these health insurers | - | - |
|  | The contract does not meet the highest contract requirements of these health insurers | - | - |

Additional file 1. Scale, items, factor loadings and Cronbach’s alpha
