# Supplementary material for: The relations between business model efficiency and novelty, and outcome while accounting for managed competition contract: a quantitative study among Dutch physiotherapy primary healthcare organisations
Source: BMC Health Serv Res. 2022 Aug 3;22:990. doi: 10.1186/s12913-022-08383-7 (PMC9351119; doi:10.1186/s12913-022-08383-7)
Supplement: Supplementary file 2 — Additional file 2. Pearson correlation for all regression variables. [file 12913_2022_8383_MOESM2_ESM.docx]

| Variable | **(1)** | **(2)** | **(3)** | **(4)** | **(5)** | **(6)** | **(7)** | **(8)** | **(9)** | **(10)** | **(11)** | **(12)** |
| --- | --- | --- | --- | --- | --- | --- | --- | --- | --- | --- | --- | --- |
| (1) Gender | - |  |  |  |  |  |  |  |  |  |  |  |
| (2) Age | -.01 | - |  |  |  |  |  |  |  |  |  |  |
| (3) Education | -.07 | -.17* | - |  |  |  |  |  |  |  |  |  |
| (4) Organisation type | .01 | -.01 | -.01 | - |  |  |  |  |  |  |  |  |
| (5) Number of departments | .04 | -.09 | .02 | .23** | - |  |  |  |  |  |  |  |
| (6) Number of employees (full time equivalent) | .12 | -.07 | -.04 | .44** | .50** | - |  |  |  |  |  |  |
| (7) Specialised therapist employed | .03 | .09 | .17 | .13 | .34** | .31** | - |  |  |  |  |  |
| (8) Managed competition contract | .11 | -.00 | .04 | .30** | .31* | .56** | .26** | - |  |  |  |  |
| (9) Business model efficiency | .01 | .11 | .15 | -.00 | .12 | .02 | .10 | .08 | - |  |  |  |
| (10) Business model novelty | .04 | -.19* | .10 | .29** | .28** | .34** | .17 | .17* | -.01 | - |  |  |
| (11) PTPHO-centred outcomes, treatment service quality | .08 | -.10 | .01 | .05 | .05 | .11 | .09 | .10 | .05 | .28** | - |  |
| (12) PTPHO-centred outcome, financial | .12 | -.10 | .11 | .01 | .11 | .28** | .27** | .09 | .02 | .29** | .45** | - |
| The numbers in the column titles refer to the variables with the same number as in the first row  * *p* <0.05; ** *p <0.01; n=138* | | | | | | | | | | | | |

Additional file 2. Pearson correlation for all regression variables
